# The 80-kb DNA duplication on BTA1 is the only remaining candidate mutation for the polled phenotype of Friesian origin

**DOI:** 10.1186/1297-9686-46-44

**Published:** 2014-07-03

**Authors:** Sophie Rothammer, Aurélien Capitan, Erik Mullaart, Doris Seichter, Ingolf Russ, Ivica Medugorac

**Affiliations:** 1 Animal Genetics and Husbandry, Ludwig-Maximilians-University Munich, Veterinärstraße 13, 80539 Munich, Germany; 2 INRA, UMR1313 Génétique Animale et Biologie Intégrative, F-78352 Jouy-en-Josas, France; 3 AgroParisTech, UMR1313 Génétique Animale et Biologie Intégrative, F-75231 Paris 05, France; 4 UNCEIA, Service Génétique, Paris, France; 5 CRV BV, P.O. Box 454, Arnhem, The Netherlands; 6 Tierzuchtforschung e.V. München, Senator-Gerauer-Straße 23, 85586 Grub, Germany

## Abstract

**Background:**

The absence of horns, called polled phenotype, is the favored trait in modern cattle husbandry. To date, polled cattle are obtained primarily by dehorning calves. Dehorning is a practice that raises animal welfare issues, which can be addressed by selecting for genetically hornless cattle. In the past 20 years, there have been many studies worldwide to identify unique genetic markers in complete association with the polled trait in cattle and recently, two different alleles at the *POLLED* locus, both resulting in the absence of horns, were reported: (1) the Celtic allele, which is responsible for the polled phenotype in most breeds and for which a single candidate mutation was detected and (2) the Friesian allele, which is responsible for the polled phenotype predominantly in the Holstein-Friesian breed and in a few other breeds, but for which five candidate mutations were identified in a 260-kb haplotype. Further studies based on genome-wide sequencing and high-density SNP (single nucleotide polymorphism) genotyping confirmed the existence of the Celtic and Friesian variants and narrowed down the causal Friesian haplotype to an interval of 145 kb.

**Results:**

Almost 6000 animals were genetically tested for the polled trait and we detected a recombinant animal which enabled us to reduce the Friesian *POLLED* haplotype to a single causal mutation, namely a 80-kb duplication. Moreover, our results clearly disagree with the recently reported perfect co-segregation of the *POLLED* mutation and a SNP at position 1 390 292 bp on bovine chromosome 1 in the Holstein-Friesian population.

**Conclusion:**

We conclude that the 80-kb duplication, as the only remaining variant within the shortened Friesian haplotype, represents the most likely causal mutation for the polled phenotype of Friesian origin.

## Background

The bovine polled phenotype, i.e. the absence of horns, has huge practical importance for breeders and is of special biological interest to geneticists. Currently, the world cattle population is estimated to be about 1.3 billion heads [1] of which a large proportion is horned. Although historical records indicate the presence of naturally polled cattle as far back as ancient Egypt [2,3], until recently, horned cattle remained the desired phenotype because it simplified tethering and attachment to harnesses. However, in modern husbandry systems, such practices are no longer used and the presence of horns increases the risk of injuries to handlers and animals, especially as the housing densities have increased. Thus, the presence of horns induces considerable economic losses in the cattle industry due to dehorning practices and the treatment of subsequent secondary infections, but also due to carcass and leather deterioration [2,4-7]. Today, dehorning of cattle at an early age is common and although records are not exhaustive, there are probably more than 100 million calves that are dehorned each year. Since all dehorning methods are invasive and thus raise animal welfare issues, the possibility of breeding genetically polled cattle is a promising alternative [4,8]. In addition, understanding the molecular mechanisms that are involved in the inhibition of the bovine horn bud differentiation will contribute knowledge on the mechanisms that underlie ectopic expression in mammals [2].

Since 1906, the polled phenotype is known to be inherited as an autosomal dominant trait [9] and the *POLLED* locus was mapped to bovine chromosome 1 (BTA1 for *Bos taurus*) in 1993 [10]. In the past years, the position of the *POLLED* locus on BTA1 was refined [11,12] and candidate causal mutations were identified [2,4,5]. Recently, Medugorac et al. [4] demonstrated the existence of at least two different alleles at the *POLLED* locus in cattle. While a complex 202-bp insertion-deletion (InDel), referred to as P_202ID_, was identified in various cattle breeds from Scandinavia, Scotland, England, the Channel Islands and France down to the Alpine region (thus also referred to as the Celtic allele P_C_), a 260-kb haplotype including five candidate mutations (three SNP: P_G1654405A_, P_C1655463T_, P_C1768587A_; two InDel: P_5ID_, P_80kbID_) was reported to be perfectly associated with the polled phenotype in the Holstein-Friesian breed (thus also referred to as the Friesian allele P_F_) [4]. Further studies based on genome-wide sequencing and high-density SNP genotyping confirmed the existence of these Celtic and Friesian variants and narrowed the causal Friesian haplotype down to an interval of 145 kb [2] that included only two of the five initial candidate mutations i.e. SNP P_C1768587A_ and InDel P_80kbID_. However, high-throughput sequencing of this 145-kb interval identified two new candidate variants i.e. SNPs P_T1764239C_ and P_G1855898A_, and genotyping of an enlarged cattle breed panel revealed the presence of the Celtic allele in new breeds from Iceland, Scandinavia, UK, Ireland and France [2]. In 2013, Allais-Bonnet et al. [2] provided evidence that introgression of the Friesian allele was ongoing in some French and German cattle breeds for which the Celtic allele was predominant and Glatzer et al. [5] reported that SNP AC000158:g.1390292G > A, hereinafter referred to as rs134968598, co-segregated perfectly with the *POLLED* locus in a set of 443 Holstein-Friesian animals. This SNP is located within intron 3 of the *interferon gamma receptor 2* (*IFNGR2*) gene and is close (1113 bp) to a SNP for which the most significant selection signature was observed in a group of polled cattle [13]. However, these two SNPs are located 378 kb from the above mentioned Friesian haplotype.

This study aimed at (1) refining the 260-kb haplotype which, based on previous results, is associated with the polled phenotype of Friesian origin [4] and (2) examining the co-segregation of SNP rs134968598 and the *POLLED* locus in Holstein-Friesian cattle. These aims are highly interesting from a practical and economic point of view for the most important global cattle breed Holstein-Friesian. Identification of the causal candidate mutation in the Friesian haplotype will also contribute to decipher the molecular mechanisms that underlie this complex developmental phenotype with allelic heterogeneity.

## Methods

### Ethical statement

No formal ethical approval was required, since no new tissue samples were collected for this study. Indeed, for most animals, the DNA samples from previous studies [4,12] were in stock in our laboratory and the remaining DNA samples were obtained from routine testing (paternity and polled phenotype test) by appropriate cattle breeding organizations.

### Animal samples

**Figure 1:**
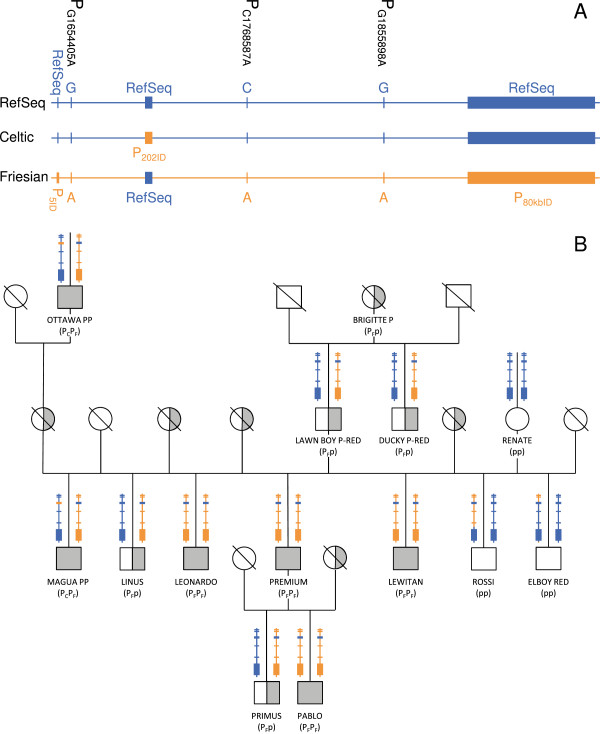
**Polled phenotype of Friesian origin. A)** Relative position of the candidate mutations for the polled phenotype of Celtic (P_C_) and Friesian (P_F_) origin. All reference sequence (RefSeq) alleles are shown in blue and *POLLED* candidate mutations in orange. The insertion-deletion events (P_5ID_, P_202ID_ and P_80kbID_) are presented as bars and SNPs as lines. Note that this figure does not include the fairly distant SNP rs134968598 at position 1 390 292 bp. **B)** Pedigree of 13 Holstein-Friesian animals used to narrow down the interval containing the *POLLED* mutation of Friesian origin. Color code is as above. The recombinant haplotypes of the ROSSI and ELBOY RED bulls are shown with the corresponding colors. Polled homozygous (PP) individuals are represented by solid circles (females) and squares (males); declared polled heterozygous animals (Pp) by half-filled symbols; horned animals (pp) by empty symbols; missing and ungenotyped individuals are marked with a diagonal line.

A total of 5993 bovine individuals were genotyped for the Celtic allele P_202ID_ and for the two InDel mutations that flank the Friesian haplotype i.e. P_5ID_ and P_80kbID_ (Figure 1A) using previously described methods [4]. A small proportion of these animals was also genotyped for the three other mutations in the Friesian haplotype i.e. (i) animals that were shown to carry both the Celtic and the Friesian alleles on two different chromosomes (~1.2%), (ii) animals that were recombinant within the Friesian haplotype (~0.02%) and (iii) a few animals that displayed an initial ambiguous genotyping result (~1.5%). To refine the Friesian haplotype, we included the two recombinant animals and all their available ancestors and relatives, which constituted a pedigree of 13 genotyped Holstein-Friesian animals (Figure 1B).

To test the previously reported [5] co-segregation of the *POLLED* locus with SNP rs134968598, which is located outside of the 260-kb haplotype, we used 130 animals that were representative of the global polled and horned Holstein-Friesian cattle population. These 130 animals included animals that we also used to refine the Friesian haplotype and bulls that had already been analyzed in our previous study [4]. Of the 130 animals, 69 were horned (pp), 54 were heterozygous polled (Pp) and seven were homozygous polled (PP). The *POLLED* genotype of each polled bull based on progeny testing was available from the respective breeding organizations. The progeny test considered phenotypes of at least nine progeny per bull mated to horned dams.

### Sequencing of the P_T1764239C_ candidate mutation

In our previous study, the region captured around the SNP P_T1764239C_, one of the two new candidate SNPs reported by [2], was sequenced by high-throughput sequencing, but the sequence coverage was low [4]. Therefore, this region was re-sequenced by conventional Sanger sequencing [14] using DNA samples from four horned animals for which, in the previous study, the sequence capture data suggested a discrepancy between the known phenotype and the expected genotype, although the coverage was too low to infer reliable genotypes.

### Refining the Friesian haplotype

In order to refine the 260-kb haplotype that perfectly associates with the *POLLED* locus of Friesian origin, we genotyped 13 Holstein-Friesian individuals (that formed a small pedigree with two recombinant bulls and 11 relatives) for the Celtic allele P_202ID_ and the four Friesian variants P_5ID_, P_G1654405A_, P_C1768587A_ and P_80kbID_ as described in Medugorac et al. [4]. Furthermore, of the two additional Friesian SNPs P_G1855898A_ and P_T1764239C_ reported in Allais-Bonnet et al. [2], we included only the candidate causal mutation P_G1855898A_ because preliminary Sanger sequencing results indicated that P_T1764239C_ segregated in horned animals. Thus, the 13 Holstein-Friesian individuals were also genotyped for P_G1855898A_ using PCR-RFLP. Therefore, PCR products of the region around P_G1855898A_ were amplified using the primers reported in [2], digested with the restriction enzyme *PstI* and the digestion products were size-separated by electrophoresis on a 2% ethidium-bromide stained agarose gel.

### Genotyping of SNP rs134968598 to test co-segregation with the *POLLED* locus

The 130 animals, including those used to refine the haplotype, were genotyped for the recently published mutation within the gene *IFNGR2* (rs134968598) as described in Glatzer et al. [5].

## Results and discussion

### Genotyping of SNP rs134968598 to test co-segregation with the *POLLED* locus

Recently, Glatzer et al. [5] reported that SNP rs134968598 co-segregated with the *POLLED* locus in a set of 443 (15 PP, 71 Pp, 357 pp) Holstein-Friesian animals [5]. In our study, genotyping of 130 animals representative of the global polled and horned Holstein-Friesian cattle population (7 PP, 54 Pp, 69 pp) for SNP rs134968598 revealed discrepancies between the progeny-based genotypes and the SNP-based genotypes at the *POLLED* locus in seven cases (see Table 1). Of the 69 genotyped horned individuals (*POLLED* genotype pp), 67 were homozygous G/G for rs134968598 as expected according to [5] but two bulls were heterozygous A/G. Of the 54 proven heterozygous polled (Pp) bulls, 51 were heterozygous A/G and three were homozygous G/G. Finally, five of the seven proven homozygous polled bulls (PP) were homozygous A/A, but two were heterozygous A/G. In conclusion, ~5% of the genotypes at SNP rs134968598 are inconsistent with the previously reported perfect co-segregation between this SNP and the *POLLED* locus in Holstein-Friesian cattle. Similar to Glatzer et al. [5], our sample of 130 animals was representative of the global Holstein-Friesian population (i.e. from Canada, the Czech Republic, Denmark, France, Germany, Italy, the Netherlands and the United States of America) and among the polled individuals that exclude SNP rs134968598, some are major breeding bulls like the homozygous polled PREMIUM PP bull which has been extensively used in recent years.

**Table 1:** **Seven bulls for which the progeny-based *POLLED *** **genotype and the genotype at SNP rs134968598 did not match**

**Breeding bull**	**International ID**	**Polled**	**rs134968598**
ELBOY RED	FRAM000109031700	pp	A/G
ROSSI	NLDM000538994750	pp	A/G
JOSEF PS	DEUM000768421954	Pp	G/G
LEGOLAS P	DEUM000352976294	Pp	G/G
PRIMUS P	DEUM000536884209	Pp	G/G
PABLO PP	DEUM000769554302	PP	A/G
PREMIUM PP	USAM000139419625	PP	A/G

### Refining the Friesian haplotype

After our genotyping data excluded the rs134968598 SNP on BTA1 at position 1 390 292 bp as a candidate mutation, we re-analysed our sequence capture data and partly re-sequenced a region around the P_T1764239C_ candidate mutation of the 145-kb haplotype of Friesian origin reported in Allais-Bonnet et al. [2]. Several horned animals were found heterozygous C/T at SNP P_T1764239C_ and thus this SNP was also excluded as a candidate mutation. In order to refine the Friesian haplotype, which contains the causal mutation for the polled trait in most Holstein-Friesian breeding lines, a small pedigree including two recombinant bulls and relatives (Figure 1B) was genotyped for the six known polymorphisms in this region P_202ID,_ P_5ID_, P_G1654405A_, P_C1768587A_, P_A1855898G_ and P_80kbID_ (for physical positions (according to UMD3.1) see Figure 1A).

ELBOY RED, a horned male offspring of LAWN BOY P-RED, is a recombinant between P_G1654405A_ and P_C1768587A,_ which excludes P_5ID_ and P_G1654405A_ as well as the fairly distant rs134968598 SNP as candidate mutations for the polled phenotype. Previously, we reported and confirmed this recombination in 20 horned offspring of ELBOY RED [2]. A second horned offspring of LAWN BOY P-RED, the recombinant bull ROSSI, carries a large region of the Friesian haplotype that is associated with the polled trait and that comprises P_5ID_, P_G1654405A_, P_C1768587A_ and P_G1855898A_ (Figure 1A and B). The genotyping data of ROSSI located the recombination event in an interval of 53 kb between P_G1855898A_ and P_80kbID_ (beginning at g.1909352) and clearly excluded P_5ID_, P_G1654405A_, P_C1768587A_ and P_G1855898A_ as candidates for the polled phenotype of Friesian origin.

Finally, based on the combined sequencing and genotyping data from our previous studies [2,4] and this study, the only remaining causal candidate within the Friesian *POLLED* haplotype is the 80-kb duplication P_80kbID_. This conclusion is inconsistent with that of Glatzer et al. [5] who reported the existence of a progeny-proven heterozygous polled Holstein-Friesian sire that carried neither the P_80kbID_ mutation nor a Celtic mutation. Unfortunately, since the identity of this bull was not provided, we could not verify these results. However, our personal experience from different previous analyses that we carried out is, that reliable genotyping of P_202ID_ and P_80kbID_ is not trivial and that the genotyping-procedure should be validated using anonymous samples. Thus, there is a possibility that the bull reported in [5] does carry either P_202ID_ or P_80kbID_. In addition, even if we had demonstrated the absence of both P_202ID_ and P_80kbID_ in the genome of this bull_,_ further analyses would still have been necessary to show that it does not carry another spontaneous mutation, which could be responsible for a dominant horn defect syndrome. Such mutations have been reported to occur and can be confused with the regular *POLLED* locus [2,15,16]. Therefore, the existence of a new candidate mutation at the *POLLED* locus of this animal should first be investigated and supported by linkage analyses on its relatives before refuting the highly probable causality of P_80kbID_ for the polled phenotype in Holstein-Friesian cattle.

## Conclusions

Based on the genotypes of almost 6000 animals, we identified two bulls with a recombination within the 260-kb Friesian haplotype and narrowed this haplotype down to an interval of 53 kb on BTA1. Moreover, the results from these two recombinant bulls and related individuals disagree with the perfect association between the *POLLED* locus and a SNP within intron 3 of the *IFNGR2* gene previously reported by Glatzer et al. [5]. Combining the exhaustive sequencing data and high-density SNP genotyping results from previous studies [2,4] and those reported here, we provide evidence that supports the P_80kbID_ mutation as the only remaining and most likely causal mutation for the polled phenotype of Friesian origin. In addition to P_80kbID_, P_202ID_ (the *POLLED* mutation of Celtic origin) segregates in some Holstein-Friesian families and sporadically combines in some homozygous polled but heterogeneous (P_C_/P_F_) animals (Figure 1B). Neither P_80kbID_ nor P_202ID_ are located in any known coding regions [2,4] but these mutations, even as single copy, result in the inhibition of horn development at a very early stage in cattle [2,4]. Therefore, future investigations of the molecular mechanisms that underlie the inhibition of horn development in cattle will enrich our knowledge not only on the mechanisms that are involved in bovine horn bud differentiation but, also, more generally, on ectopic expression in mammals.

## Competing interests

The authors declare that they have no competing interests.

## Authors’ contributions

SR contributed to molecular genetics and statistical analyses, drafted and revised the manuscript. AC provided samples, phenotyped animals, analysed the data and critically revised the manuscript. EM provided samples, phenotyped animals and contributed to the interpretation of data. DS and IR contributed to molecular genetics analyses and provided samples and phenotypes. IM designed the study and performed statistical analyses, drafted and critically reviewed the manuscript. All authors read and approved the final manuscript.

## References

[B1] Brown LR Plan B 4.0: Mobilizing to Save Civilization 2009 New York: WW Norton & Company

[B2] Allais-Bonnet A Grohs C Medugorac I Krebs S Djari A Graf A Fritz S Seichter D Baur A Russ I Bouet S Rothammer S Wahlberg P Esquerre D Hoze C Boussaha M Weiss B Thepot D Fouilloux MN Rossignol MN van Marle-Koster E Hreietharsdottir GE Barbey S Dozias D Cobo E Reverse P Catros O Marchand JL Soulas P Roy P Novel insights into the bovine polled phenotype and horn ontogenesis in Bovidae PLoS ONE 2013 8 e63512 10.1371/journal.pone.0063512 23717440 PMC3661542

[B3] Roman A L'élevage bovine en Egypte antique Bull Soc Fr Hist Méd Sci Vét 2004 3 35 45

[B4] Medugorac I Seichter D Graf A Russ I Blum H Gopel KH Rothammer S Förster M Krebs S Bovine polledness–an autosomal dominant trait with allelic heterogeneity PLoS ONE 2012 7 e39477 10.1371/journal.pone.0039477 22737241 PMC3380827

[B5] Glatzer S Merten NJ Dierks C Wöhlke A Philipp U Distl O A Single nucleotide polymorphism within the *Interferon Gamma Receptor 2* gene perfectly coincides with polledness in Holstein cattle PLoS ONE 2013 8 e67992 10.1371/journal.pone.0067992 23805331 PMC3689702

[B6] Misch LJ Duffield TF Millman ST Lissemore KD An investigation into the practices of dairy producers and veterinarians in dehorning dairy calves in Ontario Can Vet J 2007 48 1249 1254 18189045 PMC2081989

[B7] Prayaga KC Genetic options to replace dehorning in beef cattle - a review Aust J Agr Res 2007 58 1 8 10.1071/AR06044

[B8] Graf B Senn M Behavioural and physiological responses of calves to dehorning by heat cauterization with or without local anaesthesia Appl Anim Behav Sci 1999 62 153 171 10.1016/S0168-1591(98)00218-4

[B9] Spillman WJ Mendel's law in relation to animal breeding J Hered 1906 1 171 177

[B10] Georges M Drinkwater R King T Mishra A Moore SS Nielsen D Sargeant LS Sorensen A Steele MR Zhao X Womack JE Hetzel J Microsatellite mapping of a gene affecting horn development in Bos taurus Nat Genet 1993 4 206 210 10.1038/ng0693-206 8348158

[B11] Drögemuller C Wöhlke A Mömke S Distl O Fine mapping of the polled locus to a 1-Mb region on bovine chromosome 1q12 Mamm Genome 2005 16 613 620 10.1007/s00335-005-0016-0 16180143

[B12] Seichter D Russ I Rothammer S Eder J Förster M Medugorac I SNP-based association mapping of the polled gene in divergent cattle breeds Anim Genet 2012 43 595 598 10.1111/j.1365-2052.2011.02302.x 22497248

[B13] Stella A Ajmone-Marsan P Lazzari B Boettcher P Identification of selection signatures in cattle breeds selected for dairy production Genetics 2010 185 1451 1461 10.1534/genetics.110.116111 20479146 PMC2927769

[B14] Sanger F Nicklen S Coulson AR DNA sequencing with chain-terminating inhibitors Proc Natl Acad Sci USA 1977 74 5463 5467 10.1073/pnas.74.12.5463 271968 PMC431765

[B15] Capitan A Allais-Bonnet A Pinton A Marquant-Le Guienne B Le Bourhis D Grohs C Bouet S Clement L Salas-Cortes L Venot E Chaffaux S Weiss B Delpeuch A Noe G Rossignol MN Barbey S Dozias D Cobo E Barasc H Auguste A Pannetier M Deloche MC Lhuilier E Bouchez O Esquerre D Salin G Klopp C Donnadieu C Chantry-Darmon C Hayes H A 3.7 Mb deletion encompassing ZEB2 causes a novel polled and multisystemic syndrome in the progeny of a somatic mosaic bull PLoS ONE 2012 7 e49084 10.1371/journal.pone.0049084 23152852 PMC3494662

[B16] Capitan A Grohs C Weiss B Rossignol MN Reversé P Eggen A A newly described bovine type 2 scurs syndrome segregates with a frame-shift mutation in TWIST1 PLoS ONE 2011 6 e22242 10.1371/journal.pone.0022242 21814570 PMC3141036

